# Understanding the Nature of Metadata: Systematic Review

**DOI:** 10.2196/25440

**Published:** 2022-01-11

**Authors:** Hannes Ulrich, Ann-Kristin Kock-Schoppenhauer, Noemi Deppenwiese, Robert Gött, Jori Kern, Martin Lablans, Raphael W Majeed, Mark R Stöhr, Jürgen Stausberg, Julian Varghese, Martin Dugas, Josef Ingenerf

**Affiliations:** 1 IT Center for Clinical Research University of Lübeck Lübeck Germany; 2 Institute of Medical Informatics University of Lübeck Lübeck Germany; 3 Chair of Medical Informatics Friedrich-Alexander-Universität Erlangen-Nürnberg Erlangen Germany; 4 Department Epidemiology of Health Care and Community Health, Institute for Community Medicine University Medicine Greifswald Greifswald Germany; 5 Federated Information Systems German Cancer Research Center Heidelberg Germany; 6 Complex Data Processing in Medical Informatics University Medical Center Mannheim Mannheim Germany; 7 Universities of Giessen and Marburg Lung Center German Center for Lung Research Justus-Liebig-University Giessen Germany; 8 Institute of Medical Informatics University Hospital RWTH Aachen Aachen Germany; 9 Institute of Medical Informatics, Biometry and Epidemiology Faculty of Medicine, University of Duisburg-Essen Essen Germany; 10 Institute of Medical Informatics University of Münster Münster Germany; 11 Institute of Medical Informatics Heidelberg University Hospital Heidelberg Germany

**Keywords:** metadata, metadata definition, systematic review, data integration, data identification, data classification

## Abstract

**Background:**

Metadata are created to describe the corresponding data in a detailed and unambiguous way and is used for various applications in different research areas, for example, data identification and classification. However, a clear definition of metadata is crucial for further use. Unfortunately, extensive experience with the processing and management of metadata has shown that the term “metadata” and its use is not always unambiguous.

**Objective:**

This study aimed to understand the definition of metadata and the challenges resulting from metadata reuse.

**Methods:**

A systematic literature search was performed in this study following the PRISMA (Preferred Reporting Items for Systematic Reviews and Meta-Analyses) guidelines for reporting on systematic reviews. Five research questions were identified to streamline the review process, addressing metadata characteristics, metadata standards, use cases, and problems encountered. This review was preceded by a harmonization process to achieve a general understanding of the terms used.

**Results:**

The harmonization process resulted in a clear set of definitions for metadata processing focusing on data integration. The following literature review was conducted by 10 reviewers with different backgrounds and using the harmonized definitions. This study included 81 peer-reviewed papers from the last decade after applying various filtering steps to identify the most relevant papers. The 5 research questions could be answered, resulting in a broad overview of the standards, use cases, problems, and corresponding solutions for the application of metadata in different research areas.

**Conclusions:**

Metadata can be a powerful tool for identifying, describing, and processing information, but its meaningful creation is costly and challenging. This review process uncovered many standards, use cases, problems, and solutions for dealing with metadata. The presented harmonized definitions and the new schema have the potential to improve the classification and generation of metadata by creating a shared understanding of metadata and its context.

## Introduction

Computer-aided medicine is revolutionizing health care and is creating treatment possibilities that are unimaginable without computer assistance: personalized medicine, improved diagnostics by artificial intelligence, and robot-assisted surgery. An immense amount of data fuels this digital revolution, and it is desperately needed for specialized procedures to be developed and optimized. This information is primarily created to document patient care for legal or financial purposes [1] and is often stored in silos [2], consequently making it hard to reach and impossible to reuse. Owing to the missing exchange, data formats will differ, creating data heterogeneity, which is a well-discussed issue in computer science [3]. Metadata can support the integration of heterogeneous data sources to achieve a valid and meaningful data fusion, enabling a comprehensible reuse of the stored medical information [4]. Metadata are created for a detailed and unique description of the corresponding data. It serves various use cases in different research areas, for example, data identification, classification, retrieval, and data set validation. The unambiguous and precise definition of metadata is crucial and is increasingly becoming a focus of active research. An important aspect of the research is the proposed findability, accessibility, interoperability, and reusability principles by Wilkinson et al [5], which are clear guidelines for the association of data and metadata. However, from current experiences, the definition of the term “metadata” is far from clear and very nonuniformly applied in everyday life. The problem is the variety of definitions, formats, standards, and contexts, which leads to a vague understanding of the actual metadata itself. The harmonization aspect, which was intended to be solved by using metadata, resulted in another form of heterogeneity instead of a solution for missing interoperability. It appears that domain experts providing clinical metadata and metadata experts have different definitions and boundaries of 2 central metadata concepts: the definition of the metadata itself and metadata composition (like matching, mapping, and transformation). To our knowledge, there exists no analysis on these concepts found in the current literature. To close this knowledge gap, we performed an expert review using the literature from the last decade. The review’s focus and the proposed research questions were driven by the issues and misunderstanding experiences on a daily basis in our intersectoral projects. Thus, a precisely defined harmonized understanding of the term “metadata” would therefore be indispensable for current and future developments in all aspects of data integration. To ensure a wide definition of metadata, various research fields (including social science, geography, and bibliography) were investigated for metadata applications, focusing on the described problems, provided solutions, and their transferability to the field of medical informatics.

## Methods

### Design

The systematic literature review performed in this study was done following the PRISMA (Preferred Reporting Items for Systematic Reviews and Meta-Analyses) guidelines for reporting systematic reviews. A harmonization process preceded the review to gain a general understanding of the used terms.

### Harmonization Process for Quality Assurance of the Review

This review was performed by 10 reviewers with expertise in medical informatics and technical and semantic interoperability [6] of medical metadata. All reviewers had different professional backgrounds: physicians, medical computer scientists with different technical expertise, and metadata curators. Initially, we recognized a missing general use of the technical terms in the field of metadata. Therefore, to guarantee a consistent understanding of the terms among the experts and to minimize the misinterpretation and misclassification during the analysis process, the actual review was preceded by a harmonization process resulting in a joint agreement on the definitions of metadata matching, mapping, and transformation. A questionnaire was created containing 5 questions and tasks concerning the scientific background in metadata, classifications of metadata processing, and their potential for automation and the definitions of metadata matching, mapping, and transformation.

### Systematic Review

The PRISMA guidelines were applied in the systematic review as a de facto standard [7]. The process started by defining distinct and clear research questions that should be answered by the literature review. Daily work with metadata for clinical data integration has shown that there is no clear understanding of metadata and its potential applications by the users and experts. As an example, matching can be understood in various ways. Metadata matches to instance data [8] or to semantic attributes [9] or other metadata [10]. The general understanding is ambiguous. Therefore, our study aimed to explore to find an acceptable definition of metadata (Q1) and, with our operational focus on data integration, definitions for metadata processing (Q2) to enhance our daily operational tasks. In addition, we aimed to provide an overview of the variety of metadata standards used (Q3) and the generation of metadata in other research domains (Q4) to understand the issues involved and how they are solved (Q5). Thus, the focus questions were as follows:

Q1: How is the term “metadata” defined in different research fields?

Q2: How are the terms “metadata matching” and “metadata mapping” defined?

Q3: Which standards concerning metadata are in use?

Q4: How are metadata created in other research fields?

Q5: What are the current problems regarding the use of metadata, and which solutions are mentioned?

### Data Sources and Search Criteria

The review and its results were based on extensive literature analysis; therefore, the selected literature was extremely important to the results. In this review, Scopus and Web of Science was used. The selection phase was 2-fold: in the first step, the very general keyword “metadata” was used to obtain a wide variety of publications. The search query was restricted to include only journal papers, conference proceedings, and book chapters from the last 10 years (2010-2019). About 11.6% (2453/21,161) of the resulting papers were randomly selected and then analyzed by title and abstract to identify papers within the scope of the research questions. Potential publications that were of uncertain use were included at this stage to prevent hasty exclusion. The keywords of suitable papers were used in the second step of the literature search for the full-text analysis. The papers of the second literature query were analyzed by titles and abstracts again to match the research questions for inclusion in the full-text analysis.

### Review Process

Each of the 81 papers was reviewed by the first author and 2 randomly assigned reviewers, resulting in 3 independent interpretations per paper. To standardize the review process, a survey form with 8 questions was created: 6 questions corresponding to the research focus and 2 questions to gain additional information about the selected literature. The main questions focused on the metadata definitions (Q1), scoping metadata matching, mapping, and transformation (Q2), used standards (Q3), applied use cases (Q4), encountered problems, and the corresponding solutions (Q5). The additional questions covered the research field from which the paper originated and which type of metadata is described. For the categorization of the metadata types, a classification published by the National Information Standards Organization (NISO) [11] was used, which should help to classify metadata into the introduced categories better. This classification introduced 3 different types:

- descriptive metadata describe a resource for discovery and identification purposes,
- structural metadata describe the schema, data models, and reference data, and
- administrative metadata provide information about the management of a resource.

To illustrate the classification, consider this example: a book can be described using 3 different types of metadata. Author, title, and preface are examples for descriptive information, whereas the arrangement in chapters and page ordering is structural metadata. Information about the publication date and copyright information is classified as administrative metadata. The review process was open for 8 weeks. The results were gathered and analyzed by the first author and verified by the reviewers to produce a joint agreement on the final results. Both survey forms and the review results can be found in Multimedia Appendix 1 and Multimedia Appendix 2.

## Results

### Harmonized Definitions for Metadata Processing

**Figure 1:**
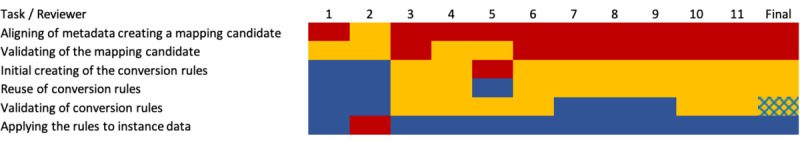
Reviewers' categorization of the tasks of a metadata-driven data integration process. Red: matching; yellow: mapping; and blue: transformation.

Ten reviewers participated in the harmonization process. The reviewers categorized 6 metadata processing tasks concerning the use case of metadata-driven data integration as matching, mapping, or transformation. Furthermore, the reviewers assessed to which degree the metadata processing tasks can be automated. The results showed a strong agreement on every task shown in Figure 1, except for the fifth task, “validation of conversion rules.” The classification “transformation” was agreed upon for conformity. Based on the results, the agreed definition for the 3 terms was created in a consensus of all 10 reviewers.

#### Matching

The matching process describes the alignment of given data structures or metadata and creates an alignment proposal between the individual data elements. These matching candidates can be created by domain experts or matching algorithms by using equivalence classes (eg, equivalent, narrower, broader).

#### Mapping

In the mapping process, a domain expert uses the proposals of the matching process to define functions or uses external rule sets (eg, Unified Code for Units of Measure) to transform the source data structure into a target data structure. The conversion functions are not necessarily symmetrical.

#### Transformation

The transformation process combines metadata and instance data. It uses the conversion rules defined in the mapping process to transform instance data according to the target data structure.

### Systematic Review

**Figure 2:**
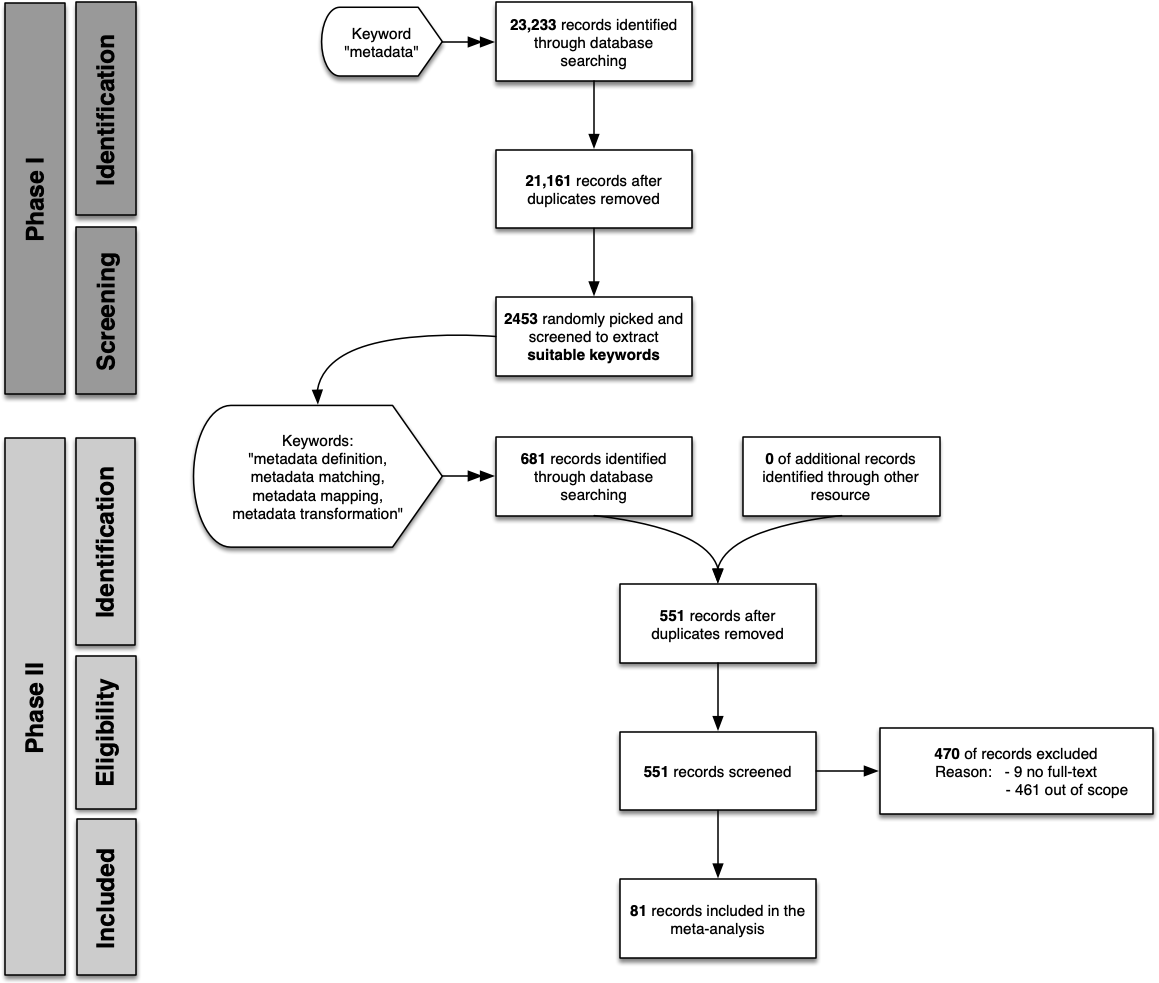
The process for literature selection in 2 search phases with different keyword sets. Two separate literature inquiries were performed: the first inquiry aimed at identifying suitable keywords for the second literature inquiry, which provided papers for the full-text analysis.

The first inquiry with the general keyword “metadata” was performed in mid-December 2019 and resulted in 23,233 papers—21,161 after duplication removal. Approximately 11.6% (2453/21,161) of the documents were randomly selected, resulting in 2453 publications whose titles and abstracts were analyzed by the first author. The keywords of the relevant papers extended the search phrase to metadata definition, metadata matching, metadata mapping, and metadata transformation. The literature search was repeated in February 2020 using the extended search phrase in the second phase, resulting in 681 papers and 551 papers after removing the duplicated entries. The titles and abstracts were analyzed to match the scope by the first author, and 81 papers were selected for the full-text analysis (Figure 2). The papers were distributed across different disciplines: medical informatics (41papers), bibliography (10 papers), bioinformatics (8 papers), informatics (8 papers), social science (8 papers), geography (4 papers), neuroinformatics (1 paper), energy informatics (1 paper), and chemistry (1 paper). The review process was open for 8 weeks. The completed PRISMA checklist can be found as Multimedia Appendix 3. The results were gathered and analyzed by the first author and then discussed and approved by the reviewers.

### Definition and Classification of Metadata

Guerra et al [12] stated that “metadata is an overloaded term in computer science and can be interpreted differently according to the context.” The literature review confirmed this ideology, and the selected publications offered a variety of definitions. However, the general notion was that metadata is a formal representation of data that defines and describes information in a (preferable) standardized and stable way [13,14]. Various characteristics of this metadata definition were extracted from the publications:

- Small atomic units describing and constraining a specific object (table fields, attributes of form questions, records) [15]
- Describes data type, range, or set of possible values [16,17]
- Single units can be composed into complex elements [18]
- Single units are often called Data Element following the International Organization for Standardization (ISO) 11179 [19]
- Metadata can have bindings to terminologies, controlled vocabularies, and taxonomies [20,21]
- Metadata repositories or data dictionaries are used synonymously and store metadata centrally [16,22-24]
- Separation of content information from layout information [17]
- Detailed machine-readable and actionable descriptions to enable data processing without human guidance [10,25]

**Figure 3:**
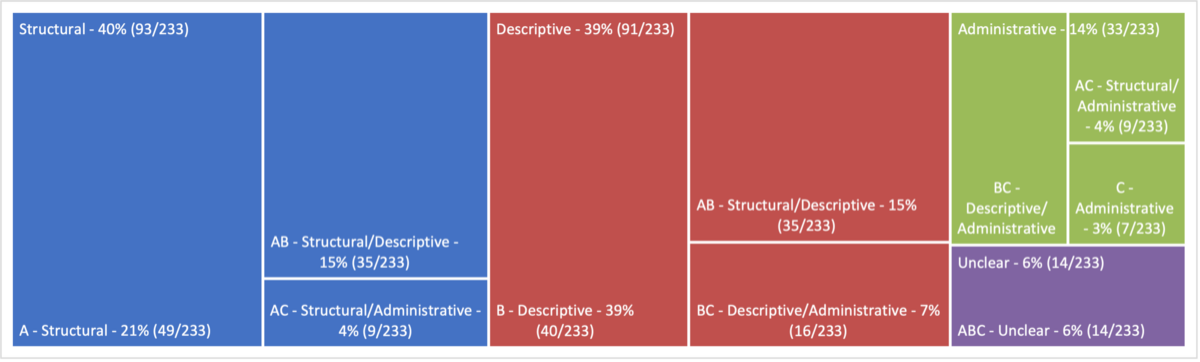
The distribution of the publications included in this review. The categories were letter-encoded: A is structural, B is descriptive, and C is administrative, as well as their resulting combination. Structural (40%) and descriptive (39%) papers were clearly in the majority, while administrative (14%) papers were rarely found. Lastly, 6% of the papers could not be clearly classified.

The NISO classification task showed that the majority of the papers were classified as structural or descriptive—papers with a pure focus on administrative metadata were a minority in the selected publications, as shown in Figure 3. The categorization of metadata according to the NISO has been described extensively elsewhere [26-29], but different definition schemes have also been encountered. Chu et al [30] introduced the separation of metadata with and without dependencies on the context. An important discriminator here is that some metadata capture information that is not dependent on the data. Context-independent metadata could describe more technical, provenance-specific records, whereas context-aware metadata could define the records descriptively to improve identification. The study from Grewe et al [31] described a new concept to annotate neurophysiological reports to capture as many annotations as possible. Therefore, the authors differed between *hard* and *soft* metadata. Parameters and information that could be directly measured (eg, temperature or timestamps) and assessed were called hard, whereas the reason of the experiment, the context information, and experiment rationale were labeled as soft metadata. Li et al [32] designed a data management system for a maritime observatory network and distinguished between 4 different metadata types: *data quality information* to ensure data reliability, *reference system information* to capture temporal and regional reference data, *maintenance information* to display updates and lifecycles, and *identification data,* which was the only mandatory type. A different categorization approach was chosen by Zozus and Bonner [33], which selected the described entity: record-level or data value–level metadata. This approach is particularly interesting for clinical studies, as the description at the value level, that is, the individual question fields in a study, is more conclusive than just a general description of the study.

### Definitions of Matching, Mapping, and Transformation

Besides descriptions of metadata representations, some authors stated their understanding of metadata matching and mapping. Ashish et al [16] defined mapping as a one-to-one relationship across 2 data elements and a set of matching candidates as a suggestion window. Rebaï et al [34] described that mapping is a semantic correspondence relation between 2 metadata schemes, which have been identified in a schema matching process. Mate et al [35] shared this definition and considers mapping candidates as the result of a matching process. If a human expert approved the relation, a mapping candidate would become a mapping. In the study from Bernstein et al [36], a new differentiation was introduced: explicit and inferred mappings. An explicit or rather direct mapping was created between 2 metadata elements, whereas an inferred mapping used explicit mapping to create new relations like a metadata crosswalk [37]. Definitions of transformation were not found in the reviewed papers except in papers in which the reviewer coauthored [35,38]. The Fleiss kappa was calculated [39] for classification on the processing task to evaluate the interrater reliability, as seen in Table 1.

**Table 1:** The Fleiss kappa values to evaluate the interrater reliability of the classification task. Values between 0.00-0.20 are classified as slight agreement and values between 0.21-0.40 as fair agreement [39].

Task	Metadata processing task		
	Matching	Mapping	Transformation
Fleiss kappa	0.13175743	0.22358548	0.29233227

### Used Metadata Standards

This review served to obtain insights into the standards and core data sets used. The assessments resulted in 37 relevant standards mentioned and used in the selected publications. The identified standards were grouped afterward into 3 categories following the levels of interoperability [40] for better oversight:

- Structure standards: ISO 11179, ISO 15926, ISO 19101, ISO 19763, ISO 20943, ISO 21526, ISO 23081, openEHR, CDISC ODM, OMOP, IHE DEX, Dublin Core, ASTM CCR, CaDSR, EAD, GILS, VRA, CIMI, CSDGM, ONIX, MARC, TMA DES, EXIF, INSPIRE, SKOS, DCAT, W3C PROV
- Technical standards: XML, RDF, OWL, JSON-LD, ClaML
- Semantic standards: ICD-10, UMLS, SNOMED CT, LOINC, MedDRA, RxNorm.

### Use Cases

Metadata are used for various use cases. The papers included in this review showed that metadata were mainly used for 4 tasks: information retrieval (21 papers), data integration (19 papers), core data set definition (10 papers), and the secondary use of data (7 papers). For information retrieval, metadata, especially semantic annotations, were used to improve query-based machine processing. Owing to a broader range of information descriptions, queries can be more accurately matched and thus, return more optional results. The processes of data integration and core data set definition used metadata to describe and harmonize the underlying schema, which can be used for secondary use of (eg, clinical) data. Further encountered use cases were an automatic data quality check [25,41] or ontology generation [42].

### Problems and the Proposed Solutions

The reviewed papers addressed several problems regarding the processing and the use of metadata in different research fields and introduced solutions with new approaches to overcome obstacles. On analyzing the papers upon with described issues, we identified 5 problem categories: (1) structural-related problems, (2) semantics-related problems, (3) human interaction–related problems, (4) metadata lifecycle–related problems, and (5) metadata processing–related problems.

#### Structural-Related Problems

According to our review, the largest group of problems were structural-related issues. The authors of the reviewed papers described a lack of standard usage. They criticized a limited or confusingly extensive selection of suitable standards [41,42]. This affected the complexity of metadata [21] and data quality [36], which led to the underutilization of metadata [36]. The absence of standards and thus, their nonuse created several problems: metadata were heterogeneous in structure and format and contained bad or missing descriptions, preventing the understanding of existing metadata and resulting in low quality [43,44]. Using different units or precision for quantitative measurements complicated the usage [27], and the heterogeneous formats prevented machine readability, which therefore worsened the identification [45,46], accessibility [47], retrieval [31], and validation [26]. However, it must be emphasized that even the constant usage of standards did not avoid heterogeneity. Current standards have no extensibility functions to be future-proof [30] nor provide modularity to compose metadata blocks from different standards [48]. The commonly used standard ISO 11179 was no exception concerning those problems: missing hierarchical or temporal dependencies [13] and missing structural [49,50] or semantic extensions [51,52]. Several improvements concerning structural issues were found in the review: reducing ISO 11179 entities to streamline and improve ease-to-use [49], reconstructing the base models [53], or establishing a supermodel integrating all proprietary extensions and adaptions of the ISO 11179 [50]. A vast selection of standards was not conducive and foments metadata heterogeneity [22]. A good example is the field of bibliography, which has too many competing standards [54]. A possible way out of this standard jungle would be to reduce their amount by only using standards accepted by the research community [17] or reusing existing and validated data elements and definitions [25,55]. If no standards were suitable or the current method for defining standards was no longer appropriate, a new conceptual approach may help. Instead of creating new standards, Woodley [37] encouraged more investment in more effort in model agreement and model reconciliation. Corradi et al [18] described the use of an event-driven model to tackle the missing extensibility. Grewe et al [31] proposed a generic metamodel approach based on 5 characteristics: extensibility, modularity, refinements, multilingualism, and machine processability.

#### Semantics-Related Problems

Semantics is a big enabler for (meta)data reuse, and therefore, according to the literature, the lack of semantics was a difficult obstacle to overcome. A general problem related to every standardized data capture was the free-text elements [56]. Metadata elements also contained descriptions and definitions to understand the purpose of the items, but these included synonyms and spelling variations or naming conflicts [44], causing a data discrepancy problem if such data were shared. A viable solution was adding semantic codes to the corresponding data elements, which represented a deeper semantic understanding. Eichenlaub et al [44] assumed that de facto standard thesauri from research fields—in the authors’ case fashion—did not cover (commonly) used terms, or the use of proprietary codes cause semantic heterogeneity [17]. The reviewed papers proposed a better annotation process, which a domain expert or natural language processing tools [23] should execute, supplemented by postcoordination and an expert review to ensure consistent encoding [57]. An essential addition would be the access and reuse of approved semantic annotations [20,58] or mapping property codes to standardized vocabularies [56].

The reviewed literature described another possible solution: the use of ontologies [15,59]. However, a problem with this approach was that an ontology must be created [60] or automatically constructed to match the instance data [61]. The reuse of existing ontologies and adaption to the custom requirements was likely a better and more adaptable choice [15]. However, problems arose when reusing ontologies owing to the metadata’s necessary conformity with the ontology structure [62].

#### Human Interaction–Related Problems

The collaboration was described as an essential aspect mentioned in the reviewed papers from each research field. Sharing and discussing the created information was not only an opportunity to improve the designed data but a necessary step to overcome the hurdles of misinterpretation [48]. Human involvement was time- and resource-consuming owing to unfamiliar or complicated software, which resulted in a low level of user acceptance [17]. Thus, metadata models or the corresponding software [63] were too complicated for health care professionals without certain necessary information technology skills [32] and therefore rarely used. In addition to the technical issues, the problem extended to the conceptual level: the model would not be clearly comprehensible if the stakeholder, users, and organizations slightly deviated in their understanding of the use cases [44]. As Varghese et al [55] aptly noted, simple disagreements about modeling decisions led to inadequate models. A tight feedback loop was recommended between users and the metadata curator to match the expected outcomes and a shared understanding of the metadata elements [44,64]. For example, extending metadata vocabularies with natural definitions would help to support the end users [64]. Nevertheless, vocabularies should be created with simplicity in mind and sufficiency instead of exhaustive description [65] as well as tooling. In the reviewed papers, 2 solutions were proposed. One approach stated that improved tools would enable medical experts for data modeling and a direct quality validation [17]. The second approach was to divide the work: the domain experts could deliver the knowledge, and metadata professionals would compose metadata in consultation, resulting in excellent and reusable metadata [66].

#### Metadata Lifecycle–Related Problems

Another vital issue is the divergence of data and the corresponding metadata [14]: data did not match the metadata and thus was not fit for reuse. The reasons for this were diverse: the lack of transparency of the (meta)data origin [47] or the boundary between data and metadata was unclear or rather a matter of changed perspective [28]. A viable approach was the extraction of metadata from the primary information technology systems and to populate it directly [23]. However, distributed metadata could vary across multiple data sources [67], and duplicates yielded the risk of staleness, particularly if the information was out-of-sync due to the extensive costs of metadata maintenance [51]. The reviewed publications state various measures that could be used against metadata staleness: continuous adaption and curation of metadata [43], tracking of changes during the metadata creation process [68], maintaining linkage information about provenance [69], and establishing a metadata lifecycle model [54]. Vos et al [70] pointed out a decisive circumstance: there is no current standard for archiving and preservation to cover the entire metadata lifecycle. However, especially archiving metadata was also the key to the reuse of archived data. Without the corresponding and descriptive metadata, the data would be difficult to reuse. Shean and Greninger [71] described that clinical metadata could even raise data privacy problems. Metadata may be used to infer other privacy-sensitive information. For example, metadata describing the parameter set specific for a HIV test connected to a particular patient could reveal the suspected disease and the diagnostic procedure to clarify the circumstances. Therefore, metadata should be considered to be anonymized before sharing to avoid data privacy concerns.

#### Metadata Processing–Related Problems

Metadata are often used for data harmonization to reduce labor. However, the process of metadata harmonization was usually performed manually [16], which was incredibly time- and resource-intensive [23]. Fortunately, the information was often machine-actionable, and therefore, automatic processing, especially matching and mapping, was possible. However, our literature review revealed known hurdles even before the metadata could be processed: heterogeneous metadata interfaces caused a siloization [72], which resulted in the impediment of metadata acquisition and reuse. If the information could be accessed, the processing also had problems: automatic matching from a broader to a more detailed level was nearly impossible [20], and if the matching results were promising, an automated mapping without human interaction was complicated or rather infeasible [16,24]. A stark problem resided in the fact that to improve the algorithms, more data for testing would be necessary, which were often challenging to obtain [38]. Moreover, the final merging of the data sets was also problematic: mappings could be ambiguous [29], the corresponding elements differed in the obligation level [73], or the proposed mapping had flaws and therefore, could cause information misinterpretation [53]. The reviewed papers proposed focusing on improving schema matching to enable a broader understanding of schemes [26]. The use of lexical and statistical methods would be enough for the matching process, and thus, the manual mapping afterward [38,57] would be indispensable to achieve adequate results. The matching could be refined with the use of unsupervised text mining techniques to calculate similarities between data elements [16]. To overcome the siloization of metadata, the use of standardized metadata search interfaces should be promoted and advanced, as shown by expanding Open Archives Initiative Protocol for Metadata Harvesting [74].

## Discussion

### Principal Results

The aim of this study was to investigate the anatomy of metadata and point out possible issues by conducting a deep insight into the recent academic literature in the last decade. It would have been desirable to extend the period to the previous 20 or even 30 years, but the amount of work would not be justifiable. The initial search for the actual review was intentionally broad with the generic key phrase “metadata,” resulting in 21,161 papers using Scopus and Web of Science. To maintain the general selection focus and minimize a self-imposed bias, domain-specific search engines such as PubMed were not used. Our selection criteria aimed for recent metadata papers with an emphasis on describing existing data sets to integrate them meaningfully. Papers dealing exclusively only with (instance) data or semantic standards were not included to reduce the immense amount of publications for review and concentrate on our core research interest. After several filtering steps, the resulting 81 papers included in the review were mainly from the field of medical informatics. This might be because metadata were very relevant to this area of research, and thus, a considerable amount of work was done in this area.

The papers’ distribution of the metadata categories was unbalanced: there were hardly any papers with an administrative orientation in the selected papers. The challenges of comprehensible data collection and traceability intensified with a substantial increase in digitization, and administrative metadata can be used to support management processes. Intriguingly, this was apparently not strongly represented in the literature. This was somewhat surprising since this information would be indispensable for the documentation of origin and traceability of data records. It appeared that the field of administrative metadata, including provenance information, has been massively underrepresented in the last decade. The use cases found were in line with our daily experiences: metadata were mainly used to improve information retrieval and data integration. Another expected facet was the sheer amount of standards (see the comparative analysis of Baek and Sugimoto [54]). The multitude of different standards leads to oversaturation and rejection, which was an essential insight for medical informatics. Consequently, awareness of a limited number of supported standards that are improved and therefore followed by the community will be an important goal.

Besides the categorization of the NISO schema, other approaches were encountered. Upon closer inspection, the newly introduced models had a considerable overlap with the schema, except for 2 approaches. Chu et al [30] emphasize the focus on the context, which was not addressed within the NISO schema. The second approach was presented by Zozus and Bonner [33], which differentiated the described information by the level of detail. As the authors stated, especially in clinical trials, the fine granular definition of the data value level would be desirable. In contrast to the bibliography, where the entire record was essential for retrieval, in clinical studies, the question level was significant and should be defined and constrained as detailed as possible.

To ensure consistency, a harmonization process preceded our review. It had to be assured that all participating reviewers had the same understanding of the definitions. This harmonization step required additional time and effort but resulted in a joint set of definitions that could be evaluated during the review. To evaluate the differences in reviewers’ understandings of these definitions, the Fleiss kappa was calculated. The results showed that the reviewers agreed on when metadata are used for mapping and transformation, although the process of matching had less agreement between experts. This can be explained by the partial mixing of the 2 definitions of matching and mapping in the analyzed publications, resulting in mixed results by the individual reviewer. The definitions and the differentiation between matching and mapping were congruent with the literature.

On the contrary, our understanding of transformation was divergent from the analyzed papers. Our definition was focusing on metadata-driven data integration: the usage of metadata for the transformation of (clinical) instance, whereas the found term *transformation* appears to be in the context of transforming the metadata itself. From this, an insight can be drawn: as a reviewer, we were influenced by our perspective on the context of metadata, and there was no consistent differentiation between *metadata transformation* and *instance data transformation*. Therefore, our definition could be used as a delimitation to define the latter field precisely.

A further important insight was the dependence on context and perspective during the definition and evaluation of metadata, as Chu et al [30] designed their new model focusing on this fact. Consistent metadata require a high level of abstraction during its creation to be generally understood by the users. This would prevent inconsistent and incorrect (re)use of the metadata and the corresponding data. A related problem is known from the field of terminology engineering using different coding systems for the postcoordination [75]. The context influences the creation of information and blurs the precise line between structure and semantics. The information that should be universally applicable in the first place is affected by an individual point of view.

### A New Schema Architecture of Metadata

**Figure 4:**
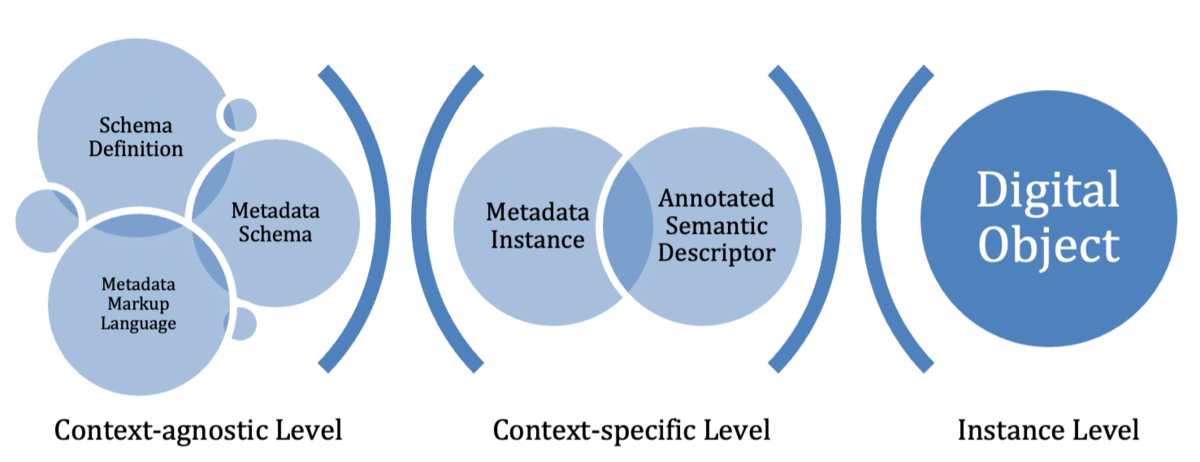
The building blocks of metadata: schema definition, metadata schema, and markup language are jointly used to instantiate metadata with an additional semantic descriptor to describe a real-world object.

Taking the decisive role of the metadata context into account, we derived a new schema for the classification of components for rich metadata objects adapting the model of Haslhofer and Klas [67]. As shown in Figure 4, the new scheme is based on the identification and separation of the context in terms of metadata creation. *Schema definition, metadata schema,* and a *metadata markup language* are context-agnostic. Representatives of each form the technical and semantic context in which a metadata object is instantiated. Metadata objects describe (non)digital objects. An illustration can be seen in Figure 3. Concerning context, the *metadata instance* can be enhanced using *annotated semantic descriptors* utilizing a variety of ontologies, terminologies, and coding systems. The instantiated metadata object can itself take the place of a digital object and be described in more detail by further metadata. This chaining mechanism allows a precise description of the highly networked nature of metadata. Further, chaining allows metadata from different systems and standards to be represented collectively in a single chained schema. An example is the enrichment of instance data with provenance information describing the origin of the metadata.

The schema definition can specify how metadata models are constructed. Well-known representatives are the norms ISO 11179 [19], ISO 15926 [76], and ISO 19763 [77]. The metadata schema describes the metadata objects with every needed attribute and is mostly the result of metadata harmonization and core data set creation, for example, Dublin Core or CaDSR. Metadata markup languages such as XML, RDF, or OWL are used for the technical description of the defined schema. The metadata schema and the metadata markup language are essential for metadata instantiation; the superimposed schema definition is not obligatory but highly recommended for comparability and interoperability.

### Limitations

This review showed that the term metadata *representation* is used as a synonym to the word *definition,* which could impact the analyzed paper selection. Furthermore, the initial paper selection could be a biased selection since the first author has a medical informatics background and was looking for a certain scope known from this. In addition, domain-specific search engines (such as PubMed) were not used; yet, the majority of papers were from the field of medical informatics. To avoid this bias, the initial selection could have been performed by various reviewers, but the sheer amount of work made this infeasible. It must also be mentioned that 10 papers were reviewed by only 2 persons because 1 reviewer had time constraints.

### Comparison With Prior Work

To our knowledge, there is no comparable systematic review of metadata processing, which includes the analysis of approved solutions from other research fields and applicability to the field of medical informatics. Nevertheless, reviews on metadata have been carried out. Baek and Sugimoto [54] produced a review, which was included in our study, on existing metadata standards used in the bibliography community to identify the most suitable standard for electronic records. This review was limited to bibliography standards but gives an impressive overview. Singh and Bawa [78] analyzed techniques for metadata management and distribution in a large-scale storage system. This review focused only on the technical or administrative aspects of metadata. The newly introduced building block schema was adapted from Haslhofer and Klas [67], and additionally, the work from Ngouongo et al [53] must be mentioned. The study classified existing metadata formats to give a comparative overview and identify the most suitable candidate for the health care sector.

### Conclusions

Metadata can be a powerful means to identify, describe, and process information, although its meaningful definition is challenging and entails significant hurdles. Different understanding of the same metadata representations is troublesome and hinders the correct utilization of metadata as well as the corresponding data instance. Through this work, 10 experts have gone through a consultation phase that ended in harmonized definitions for metadata in terms of metadata-driven data integration. This review process discovered many standards, use cases, problems, and solutions in dealing with metadata, providing a broad overview of the topic. This summary has led us to introduce a new schema for the classification of components for enriched metadata objects, which explicitly focuses on the creation context of metadata. These harmonized definitions and the new schema will improve the classification and creation of metadata by providing a mutual understanding of the metadata and its context.

## Appendix

Multimedia Appendix 1 The first survey form used for the harmonization process before the review.

Multimedia Appendix 2 The second survey form used for the actual review process.

Multimedia Appendix 3 The completed PRISMA (Preferred Reporting Items for Systematic Reviews and Meta-Analyses) checklist for the review.

## Abbreviations

ISO: International Organization for Standardization
NISO: National Information Standards Organization
PRISMA: Preferred Reporting Items for Systematic Reviews and Meta-Analyses
