# Supplementary material for: Understanding the Nature of Metadata: Systematic Review
Source: J Med Internet Res. 2022 Jan 11;24(1):e25440. doi: 10.2196/25440 (PMC8790684; doi:10.2196/25440)
Supplement: Multimedia Appendix 1 [file jmir_v24i1e25440_app1.pdf]

Systematic Review Part 1: The reviewers and their definition of metadata

The definition of metadata is to be examined in a systematic review. This survey is a structured questionnaire for the first round of the review. The invited reviewers should state their scientific background in the field of metadata processing and specify their definition conception of metadata matching and mapping. The result of the first round is a balloted definition of metadata matching and mapping.
There are 5 questions in this survey.

Rate your research focus using the three categories of heterogeneity following the IEEE definition:

> The ability of two or more systems or components to exchange information and to use the information that has been exchanged.

- Technical: using the same communication protocol to **exchange** the information
- Syntactic: using the same data format using to **read** the information
- Semantic: using the same coding system to **understand** the information

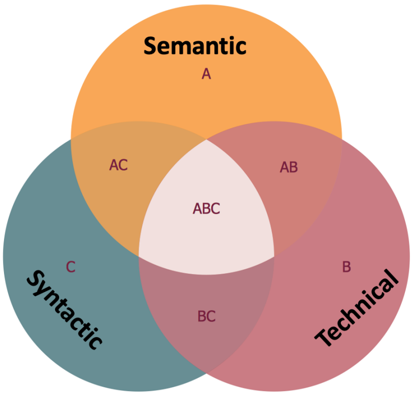

*

Please choose **only one** of the following:

- ◯ ABC - All-rounder
- ◯ A - Semantic
- ◯ B - Technical
- ◯ C - Syntactic
- ◯ AB - Semantic/Technical
- ◯ AC - Semantic/Syntactic
- ◯ BC - Technical/Syntactic

Estimate your personal research time in the field of metadata processing *

Please choose **only one** of the following:

- 0-3 years
- 3-5 years
- 5-10 years
- 10+ years
- No experience

Classify the following statements to the corresponding categories: *matching*, *mapping* and *transformation*.

*

Please choose the appropriate response for each item:

	Matching	Mapping	Transformation
**Aligning of metadata creating a mapping candidate**	○	○	○
**Validating of the mapping candidate**	○	○	○
**Initial creating of the conversion rules**	○	○	○
**Reuse of conversion rules**	○	○	○
**Validating of conversion rules**	○	○	○
**Applying the rules to instance data**	○	○	○

Rate the six tasks according to their automatability.

- **Human expert only:** the task is too dificult only human experts can perform this.
- **Semi-automatic:** the human expert is supported by algorithms.
- **Fully-automatic:** the algorithm can solve this task without human interaction.

*

Please choose the appropriate response for each item:

	Human expert only	Semi-automatic	Fully automatic
**Aligning of metadata creating a mapping candidate**	○	○	○
**Validating of the mapping candidate**	○	○	○
**Initial creating of the conversion/transformation rules**	○	○	○
**Reuse of conversion/transformation rules**	○	○	○
**Validating of conversion/transformation rules**	○	○	○
**Applying the rules to instance data**	○	○	○

An example: a local biobank wants to connect to a European-wide search network to enable its samples to be found within the network. For the data integration process, metadata is extracted from the local biobank and shall be mapped to the harmonized metadata of the network. The two given metadata sets shall be matched, mapped, and the transformation process enabled through conversion rules.

Please rate the following definitions of matching, mapping, and transformation according to your understanding of meta-driven data integration.

1. Definition according FHIR R4 4.0.1:

*Matching***:** In a ConceptMap each mapping for a concept from the source to target system includes an equivalence property that specifies how similar the mapping is (or, in some cases, that there is no valid mapping). There is one element for each concept or field in the source value set or system that needs to be mapped. Each source concept may have multiple targets:

- because there are multiple possible mappings (e.g., ambiguous)
- to specify a correct map, and specify other mappings as invalid
- when there are multiple mappings depending on the values of other elements (dependsOn)

*Mapping***:** StructureMap resource defines a detailed set of rules that describe how one Structure is related to another and provides sufficient detail to allow for automated conversion of instances.
The intention of the structure map resource is to allow a specialist in formats and interoperability to specify the full relationships between two structures (e.g. a CDA document and a set of FHIR resources), and then many different systems - both testing and production clinical systems - can leverage that to automatically transform from one format to the other. Maps are unidirectional: they map from the source structure to the target structure, and no reverse map is implied. Even if the mapping is simple, and loss-less, it cannot

be assumed that there are no conditions that might additionally apply in the reverse direction.

2. Definition according ISO/CEN 21526:

Mapping: “Map is a metaclass each instance of which represents a map between sets of administered items.” Maps contain a MapType. Map_Type is an enumerated type, each enumeration describes a kind of relationship between administered items.

3. Definition according Mate et al.:

Matching: The matching process describes the alignment of given structures or metadata and creates a mapping candidate between the individual elements.

Mapping: The mapping is a mapping candidate validated by domain expert or a matching algorithm creating conversion rules.

Transformation: The transformation process combines metadata and instance data:
using the established conversion rules to transform the instance data into the target structure.

4. Definition according Naumann et al.:

Matching: Matching creates automatically correspondence between schemes.

Mapping: (High-Level) Mapping involves two schemes and a set of correspondence. Low-Level or logical mapping is the translation of mapping according to obey the integrity conditions of both schemes and reflects the intention of the user.

Transformation: Low-level mapping is used to generate a transformation.

5. Definition according Ulrich et al.:

Matching: The matching process describes the alignment of given structures or metadata and creates an alignment

proposal between the individual elements. Matchings can be created by domain experts or matching algorithms using equivalence classes (e.g. equivalent, narrower, broader…).

Mapping: In the mapping process a domain-expert uses the proposals to define functions or uses external rules sets (e.g. UCUM) to transform the source structure into a target structure. The conversion functions are not mandatorily symmetrical.

Transformation: The transformation process combines metadata and instance data:
using the established conversion rules to transform the instance data into the target structure.

*

Please choose the appropriate response for each item:

	FHIR R4	ISO 21526	Mate et al.	Naumann et al.	Ulrich et al.
**Matching**	○	○	○	○	○
**Mapping**	○	○	○	○	○
**Transformation**	○	○	○	○	○

Submit your survey.
Thank you for completing this survey.
