# Supplementary material for: Understanding the Nature of Metadata: Systematic Review
Source: J Med Internet Res. 2022 Jan 11;24(1):e25440. doi: 10.2196/25440 (PMC8790684; doi:10.2196/25440)
Supplement: Multimedia Appendix 2 [file jmir_v24i1e25440_app2.pdf]

100%

Review

This questionaire shall be used to systematically review the given paper. In order to use the same understanding, the reviewer agreed on a harmonized definition of the terms ***matching***, ***mapping*** and ***transformation***.

Matching: The matching process describes the alignment of given structures or metadata and creates an alignment proposal between the individual elements. Matchings can be created by domain experts or matching algorithms using equivalence classes (e.g. equivalent, narrower, broader...).

Mapping: In the mapping process a domain-expert uses the alignment proposals to define **functions** or uses external rules sets (e.g. UCUM) to transform instances of the source structure into a target structure. The conversion functions are not mandatorily symmetrical.

Transformation: The transformation process combines metadata and instance data: using the established conversion rules to transform the instance data into the target structure or format.

✱Choose the corresponding bibtex key!

❗ Choose one of the following answers

Please choose...

✱Which research field is the paper from? If various categories are suitable, please choose the most content-wise relevant category!

❗ Choose one of the following answers

- ◯ Medical informatics
- ◯ Bioinformatics
- ◯ Social Sciences

Geography

Chemistry

Bibliography

Other:

✱The process of metadata-driven data transformation includes several steps: given two metadata structures, a human expert or an algorithm tries to find correspondences in the two sets and creates mapping candidates (1). The mapping candidates are validated and from the list of potential correspondences, the most suitable is/are chosen (2). In the mapping process, the validated candidates are used to create a conversion function to transform instances of the given structure from the source into the target format. Hereby either a rule is created (1) or existing rules sets, e.g., UCUM, are used (2). The created rules were qualitatively checked and released (3) to complete the mapping process. After the quality assurance process, the rules are used by a transformation engine to convert instance data of the source structure into the desired target structure/format (1).

Matching:

1. Aligning of metadata creating a mapping candidate
2. Validating of the mapping candidate

Mapping:

1. Initial creating of the conversion rules
2. Reuse of conversion rules
3. Validating of conversion rules

Transformation:

1. Applying the rules to instance data

Which category can be assigned to the paper? Which of the following tasks are described or performed in the paper?

❗ Check all that apply

Matching

Mapping

Transformation

Other:

✱How does the authors define metadata?

✱What type of metadata is described in the paper?

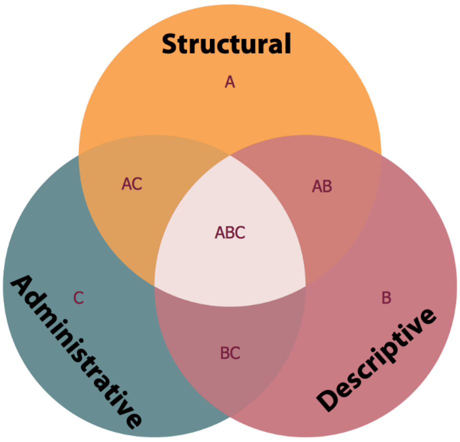

- **Structural** metadata describe, for example, data models and reference data.
- **Descriptive** metadata describe a resource for discovery and identification purposes.
- **Administrative** metadata provides information about the management of a resource.

(https://www.youtube.com/watch?v=L0vOg18ncWE&t=)As an addtional information, this video (https://www.youtube.com/watch?v=L0vOg18ncWE&t=) explains the different types of metadata in an illustrative manner.

Choose one of the following answers

Please choose...

✱Which usecase (core data set definition, data integration...) do the authors describe the use of metadata?

✱Was a standard used in the paper?

❗ Choose one of the following answers

◯ No

◯ Yes, which?

✱What problems have the authors described in dealing with metadata?

✱What solutions do the authors describe for the problems mentioned?
